# An analysis of the HIV testing cascade of a group of HIV-exposed infants from birth to 18 months in peri-urban Khayelitsha, South Africa

**DOI:** 10.1371/journal.pone.0262518

**Published:** 2022-01-14

**Authors:** Aurelie Kennedy Nelson, Tali Cassidy, Laura Trivino Duran, Vivian Cox, Catherine J. Wedderburn, Janet Giddy, Pauline Pieters, Mark F. Cotton, Tabitha Mutseyekwa, Bulelwa Rorwana, Beryl Sibanda, Jonathan Bernheimer, Nopinky Matise, Petros Isaakidis, Jean Maritz

**Affiliations:** 1 Médecins Sans Frontières, Cape Town, South Africa; 2 Division of Public Health Medicine, School of Public Health and Family Medicine, University of Cape Town, Cape Town, South Africa; 3 Desmond Tutu TB Centre, Department of Paediatrics and Child Health, Faculty of Medicine and Health Sciences, Stellenbosch University, Cape Town, South Africa; 4 Department of Paediatrics and Child Health, Red Cross War Memorial Children’s Hospital, University of Cape Town, Cape Town, South Africa; 5 Department of Clinical Research, London School of Hygiene & Tropical Medicine, London, United Kingdom; 6 Khayelitsha & Eastern Sub-Structure, Western Cape Provincial Department of Health, Cape Town, South Africa; 7 Family Center for Research with Ubuntu, Department of Paediatrics and Child Health, Stellenbosch University, Tygerberg Children’s Hospital, Cape Town, South Africa; 8 Southern African Medical Unit, Cape Town, South Africa; 9 Division of Medical Virology, Department of Pathology, Stellenbosch University, Cape Town, South Africa; 10 PathCare Reference Laboratory, Cape Town, South Africa; University of North Carolina at Chapel Hill, UNITED STATES

## Abstract

**Background:**

Despite the reduction of HIV mother-to-child transmission, there are concerns regarding transmission rate in the breastfeeding period. We describe the routine uptake of 6 or 10 (6/10) weeks, 9 months and 18 months testing, with and without tracing, in a cohort of infants who received HIV PCR testing at birth (birth PCR) (with and without point of care (POC) testing) in a peri-urban primary health care setting in Khayelitsha, South Africa.

**Methods:**

In this cohort study conducted between November 2014 and February 2018, HIV-positive mothers and their HIV-exposed babies were recruited at birth and all babies were tested with birth PCR. Results of routine 6/10 weeks PCR, 9 months and 18 months testing were followed up by a patient tracer. We compared testing at 6/10 weeks with a subgroup from historical cohort who was not tested with birth PCR.

**Results:**

We found that the uptake of 6/10 weeks testing was 77%, compared to 82% with tracing. When including all infants in the cascade and comparing to a historical cohort without birth testing, we found that infants who tested a birth were 22% more likely to have a 6/10 weeks test compared to those not tested at birth. There was no significant difference between the uptake of 6/10 weeks testing after birth PCR POC versus birth PCR testing without POC. Uptake of 9 months and 18 months testing was 39% and 24% respectively. With intense tracing efforts, uptake increased to 45% and 34% respectively.

**Conclusion:**

Uptake of HIV testing for HIV-exposed uninfected infants in the first 18 months of life shows good completion of the 6/10 weeks PCR but suboptimal uptake of HIV testing at 9 months and 18 months, despite tracing efforts. Birth PCR testing did not negatively affect uptake of the 6/10 weeks HIV test compared to no birth PCR testing.

## Introduction

Globally, there were 160,000 new paediatric HIV infections in 2018, 120,000 fewer than in 2010—a 41% reduction. Since the introduction of life-long ART irrespective of CD4 count, mother-to-child transmission (MTCT) in South Africa fell from 16,000 (2010) to 2,600 (2018) new paediatric infections [1]. To improve early diagnosis and timely initiation of antiretroviral therapy (ART) in the first few weeks of life, the World Health Organization (WHO) recommended in 2016 that, in certain contexts, birth PCR (diagnostic HIV DNA PCR at the time of birth) should be added to the standard testing at 6 or 10 weeks of age [2]. In its effort to reach the last mile for elimination of MTCT (eMTCT), South Africa adopted birth PCR for HIV-exposed infants as standard of care in 2016. Of note, the cost effectiveness of doing both a birth PCR and 10 weeks PCR, which is the current practice in South Africa, rests on the assumption that >63% of infants tested negative at birth will be brought in again for repeat testing at 10 weeks of age (10 week PCR) [3]. In most of sub-Saharan Africa, the interval between HIV early infant testing and ART initiation can be two to three months, leading to high loss to follow-up and increased infant mortality [4]. Point of care (POC) PCR for birth testing is a public health priority as it has the potential to lead to faster turnaround time [5] and to earlier ART initiation [6], despite the risk that birth POC PCR might decrease return for testing at 6 or 10 weeks [7].

Recent evidence shows that while MTCT used to occur mostly intrapartum, transmission is now either antenatal or postnatal [8–10]. Given the prolonged risk of transmission to the infant, regular testing throughout the postnatal period in breastfeeding infants is critical. It is concerning to see the estimated 20% uptake of HIV testing for children at 18 months, likely leading to very late diagnosis and delayed initiation of antiretroviral therapy [11]. A potential solution to retain exposed babies in care for regular HIV testing is to trace their caregivers. Tracing HIV patients who miss appointments is not novel and can be done in many different ways, using a variety of lay, community, home-based and health care workers [12]. In South Africa, there is such a tracing system (mostly using community health care workers) embedded within the health system [13].Some studies have suggested a high return to care of disengaged HIV and TB patients after tracing (as high as 86% returning to clinic after being traced) [14–16].

In this paper, we describe the uptake of 6 or 10 weeks, 9 months and 18 months HIV testing, with and without tracing, in a cohort of infants who received birth PCR (with and without POC testing) in a primary care setting in South Africa.

## Materials and methods

### Study design

This was a retrospective cohort study conducted between November 2014 and February 2018 in a primary care clinic in Khayelitsha.

### Study setting

Khayelitsha is a peri-urban informal settlement close to Cape Town, South Africa, with an estimated population of 500,000 and a high HIV prevalence (33.1% antenatal prevalence -2015) [17]. In Khayelitsha, mother-to-child transmission (MTCT) rate was 0.8% at 10 weeks and is unknown at 9 months and 18 months, although 18 months rapid test uptake is estimated at 34% [18]. The initial recruitment for this study took place in a large MOU (Midwife Obstetric Unit)—primary care clinic- in Khayelitsha. Follow-up of infants took place in all primary health care clinics in Khayelitsha and in one large clinic in Mfuleni, an adjacent area.

Local prevention of MTCT (PMTCT) guidelines changed during the study period: in June 2014, provincial PMTCT guidelines recommended birth PCR testing and dual post-exposure prophylaxis (PEP) for HIV-exposed infants at high risk of vertical transmission [19]. In December 2015, recommendations changed to include birth PCR for all HIV-exposed infants and to postpone the 6 weeks PCR to 10 weeks for all HIV-exposed infants [20].

### Participants and recruitment

Participants in the study were recruited at the MOU. Inclusion criteria were: all HIV-infected pregnant women (known to be infected at the time of delivery) above 18 years of age and their HIV-exposed infants, born in one primary care clinic between November 2014 and July 2016 and who consented to the study. Exclusion criteria were women-infant pairs who required imminent transfer out to a secondary care level hospital. Two full time study nurses working alternate shifts, allowed postnatal uninterrupted recruitment.

### Study procedures

#### Birth PCR

All recruited HIV-exposed newborns were offered birth HIV PCR testing. From November 2014 to July 2015, a blood sample was collected by the study nurse and sent for HIV PCR testing at the National Health Laboratory Service (NHLS) virology laboratory at Tygerberg Hospital, Cape Town, using laboratory-based Roche Cobas AmpliPrep/Cobas TaqMan (CAP/CTM) HIV-1 qualitative assay [Roche diagnostics, Branchburg, New Jersey, USA]). From July 2015 to December 2016, HIV PCR was performed in the labour ward by study nurses with POC (Alere Q 1/2 Detect [Alere Technologies GmbH, Jena, Germany], in addition to PCR testing at NHLS. From January 2016, following the changes in local PMTCT guidelines, Department of Health (DOH) nurses would collect blood sample to send for PCR testing at NHLS as part of standard of care [21].

Birth PCR samples were collected and tested in the laboratory and on POC according to methods described in S1 File. Results of birth PCR were communicated to the mothers of the infants by the study nurses. Actions taken following positive and indeterminate results are described in S1 File. Negative results on POC were communicated to the mothers as soon as they were available on the same day. Laboratory-based negative results were communicated to the mothers when they would come back to the primary care clinic for their first postnatal care visit, within six days after birth, by the study nurses. In both instances, the study nurse would counsel the patient on the need for further infant testing, infant prophylaxis and give her a date for the 6 or 10 weeks PCR test as per standard of care (provincial PMTCT guidelines changed during the study and the 6 weeks PCR was moved to 10 weeks). Mothers did let the study nurse know where they would bring their babies for follow-up HIV testing.

#### Follow-up of HIV-uninfected exposed babies and tracing

*6/10 weeks follow-up*. A full time patient tracer checked the NHLS database for evidence of PCR having been done. If no PCR was done within a month of its due date, the participant was traced.

*9 months and 18 months follow-up*. A study patient tracer went to the pre-defined “baby wellness” clinic to check the results of 9 months and 18 months rapid tests. As per standard of care, the rapid tests used were the Alere Determine tests on saliva, as per the Provincial Department of Health Guidelines [20]. If results were not found at the clinic (in the facility-held record or PMTCT and HIV testing registers) or on the electronic systems (PREHMIS, PHCIS), participants were traced.

*Tracing*. Tracing consisted of calling the mother twice on her cell phone over a period of approximately two months to find out if the infant had been taken for testing and if not, to remind her to take the child. If the phone calls were unsuccessful, a home visit was conducted by the patient tracer once and the outcomes documented for internal purposes. No additional interim contact was made with the caregiver in between the 6/10 weeks test and the 9 months test; nor between the 9 months and 18 months test. Most children were traced except if no phone numbers available.

### Data collection

#### Baseline

Study nurses completed case report forms (CRF) by reviewing folders and interviewing the mother at birth (S2 File). Data extracted included maternal and infant characteristics such as demographics, delivery details, HIV history, feeding intention, social circumstances and disclosure information. This data was entered into Epidata version v2.2 (EpiData Association, Odense, Denmark) by a trained data capturer [22]. Mother-infant dyads not receiving birth PCR nor tracing were used as a historical control group (August 2013-March 2014). Their data was collected from the labour ward register (mother’s name, folder number, date of birth and infant’s name and date of birth).

#### Follow-ups

A tracer and trained data capturer collected HIV testing data from a variety of sources: NHLS database, electronic clinic records (eKapa, PREHMIS), facility-based paper records, and PMTCT registers, used in follow-up clinics. For historical controls, 6 weeks PCR results were collected from NHLS database only. This data was entered into REDCap [23].

We refer to 6/10 weeks PCR test as a PCR test done between the ages of 6–12 weeks. The 9 months test was defined as an HIV rapid test done at 9 months of age ±30 days. The 9 months test was considered positive only if a corresponding positive PCR was recorded on the NHLS database. The 18 months test was defined as an HIV rapid test done between 17 and 19 months and considered positive if a repeat rapid HIV test was recorded as positive in the file. If no evidence of testing was found, the participant was traced, as described above.

Outcomes of the tracing were based on the information found by the patient tracer and from the information provided telephonically by the mother. Infants were considered transferred out (TFO) if they had moved outside Khayelitsha, with the exception of those receiving care in Mfuleni because of its proximity to the recruitment site. Deaths were ascertained from clinical records or if reported so by the primary caregiver. If unable to reach the mother or if the infant did not come for a test after tracing efforts, the infant was considered lost to follow-up (LTFU) a month after the last tracing intervention.

At the end of the study period, the patient tracer checked for positive PCR results (or for proxy of HIV positive results e.g. CD4 count or HIV viral load) on the NHLS database for every included infant less than 24 months of age.

### Statistical analyses

Data were analysed using Stata 14 (StataCorp (2015) Statistical Software: Release 14. College Station, Texas StataCorp LP). Descriptive statistics are presented for baseline and to describe the infant testing cascade until 18 months. Risk differences and 95% confidence intervals (CI) were calculated for testing at 6 weeks, 9 months and 18 months, by baseline characteristics, for those with available baseline data who had not yet tested positive or transferred out by each time point

#### Sub-analysis looking at retention in care for the 6/10 weeks PCR and description of historical controls

We compared two different groups of infants born at the same clinic. The first group included infants, recruited into the study, born between November 2014 to July 2016, who had laboratory-based or POC PCR. The second group was a historical control group with the following inclusion criteria: all HIV-infected pregnant women (known to be infected at the time of delivery) above 18 years of age and their HIV-exposed infants, born in one primary care clinic between August 2013 and March 2014. Of note, between August 2013 to March 2014, birth PCR at a primary care clinic was not recommended by local PMTCT guidelines and would have only been done in a research setting. For this analysis, transfer outs were not excluded in either group, as we were unable to ascertain transfer out status for the historical controls.

### Ethics

This study was conducted according to good clinical practices and the Declaration of Helsinki, with the approval of Stellenbosch University Ethics committee (HREC N14/06/060), the Western Cape Provincial Health Research Committee and the MSF-Ethics Review Board. Written consent was obtained from all mothers, for themselves and their infants.

## Results

### Baseline characteristics of participants

From November 2014 to July 2016, we recruited 781 mothers. The baseline characteristics of the mothers and infants are described in Table 1. The mean gestational age at first antenatal visit was 19 weeks and the median maternal age at delivery was 28.6 years old. Six percent of women had never attended antenatal care until delivery. Eighteen women tested positive for syphilis at their first antenatal visit; of those with known syphilis status, 12 were fully treated and 3 received 2 of the 3 treatment doses.

**Table 1:** Baseline characteristics of participants in pregnancy and in labour.

		Median (IQR)	n (%)
**At first antenatal visit**	**Never attended antenatal care**		46
	**Gravidity (N = 775)**	2 (2–3)	
	**Parity (N = 776)**	1 (1–2)	
	**Maternal age (N = 781)**	28.6 (25.1–32.7)	
	**Gestational age (N = 761)**	19 (13–25)	
	**RPR status (N = 781)**		
	Negative		718 (92%)
	Positive		18 (2%)
	Unknown		45 (6%)
	**Treatment for positive RPR (N = 18)**		
	Fully treated		10 (56%)
	Not treated		2 (11%)
	Partially treated		3 (17%)
	Unknown		3(17%)
	**HIV results at first antenatal visit (N = 781)**		
	Newly positive		223 (29%)
	Negative at first antenatal visit		9 (1%)
	Missing data		172 (22%)
	Known HIV positive		377 (48%)
	**Of the known HIV positive:**		
	On first line ARV (on NNRTI)		165 (43%)
	On second line ARV (regimen including Alluvia®)		8 (2%)
	On ART (unknown regimen)		2 (0.5%)
	Not on ART		5 (1%)
	Unknown		197 (52%)
**Labour**	**Gestational age (N = 703)**	39 (38–40)	
	**Baby birth weight (g)**	3060 (2810–3370)	781
	Mother first HIV test positive** **in labour (N = 781)**		
	No		760 (97%)
	Yes		20 (3%)
	Unknown		1 (0%)
	**Disclosure of HIV status (N = 781)**		
	To anyone		708(91%)
	To partner		471 (60%)
	No		72 (9%)
	Unknown		1 (0%)
**Risk factors**			
	**Mother initiated ART <12 weeks before delivery (N = 781)**		
	No		686 (87%)
	Yes		94 (12%)
	Unknown		1 (0%)
	**Mother diagnosed >28 weeks gestation/in labour/early postpartum (N = 781)**		
	No		760 (97%)
	Yes		20 (3%)
	Unknown		1 (0%)
	**Mother missing >1 month ART (N = 781)**		
	No		549 (90%)
	Yes		58 (10%)
	Unknown		174 (22%)
	**Mother with TB or Syphilis in pregnancy (N = 781)**		
	No		753 (96%)
	Yes		26 (3%)
	Unknown		2 (0%)
	**Baby less than 2.5kg or/and less than 37 weeks (N = 781)**		
	No		705 (90%)
	Yes		73 (9%)
	Unknown		3 (0%)
	**Maternal VL >1000 from 28 weeks in pregnancy (N = 781)**		
	No		681 (87%)
	Yes		44 (6%)
	Unknown		56 (7%)
**Total high risk mother infants (N = 781)** ***			
	No		458 (59%)
	Yes		323 (41%)

*Different N due to missing data

**First HIV positive test in labour: previous recorded HIV test was negative and had positive HIV test (screening and confirmatory positive tests) at delivery

*** High risk mother infants are mothers or infants who have at least one of the risk factors described in this table, as per the provincial definition of high risk infants at the time [20].

At the first antenatal visit, 29% of women tested HIV positive for the first time and 36% were ART-naïve. During the pregnancy, 87% of women had a viral load (VL) <1000 RNA copies/ml. Of the 607 women with available data, 10% reported missing ART for more than a month. Based on the Western Cape Province definitions [21], 51% were high risk mother infant pairs.

### Influence of birth PCR on uptake of 6/10 weeks PCR

Eighty percent of infants came back after a birth PCR for their 6/10 weeks PCR (Table 2). In the historical control group with no birth testing, 59% returned for their 6 weeks PCR (risk difference: 22%; 95%CI: 28%-16%).

**Table 2:** Comparison of percentage of infants completing 6/10 weeks HIV PCR after birth PCR or no birth PCR.

	Study infants	Historical controls
**N (excluding positive birth PCR)**	777	321
**Birth PCR**	Laboratory-based+/- POC	None
**Dates**	November 2014-June 2016	August 2013–March 2014
**% completing 6/10 weeks PCR**	80% (624/777)	59% (188/321)

### Uptake of 6/10 weeks, 9 months and 18 months HIV test (before and after tracing)

**Fig 1:**
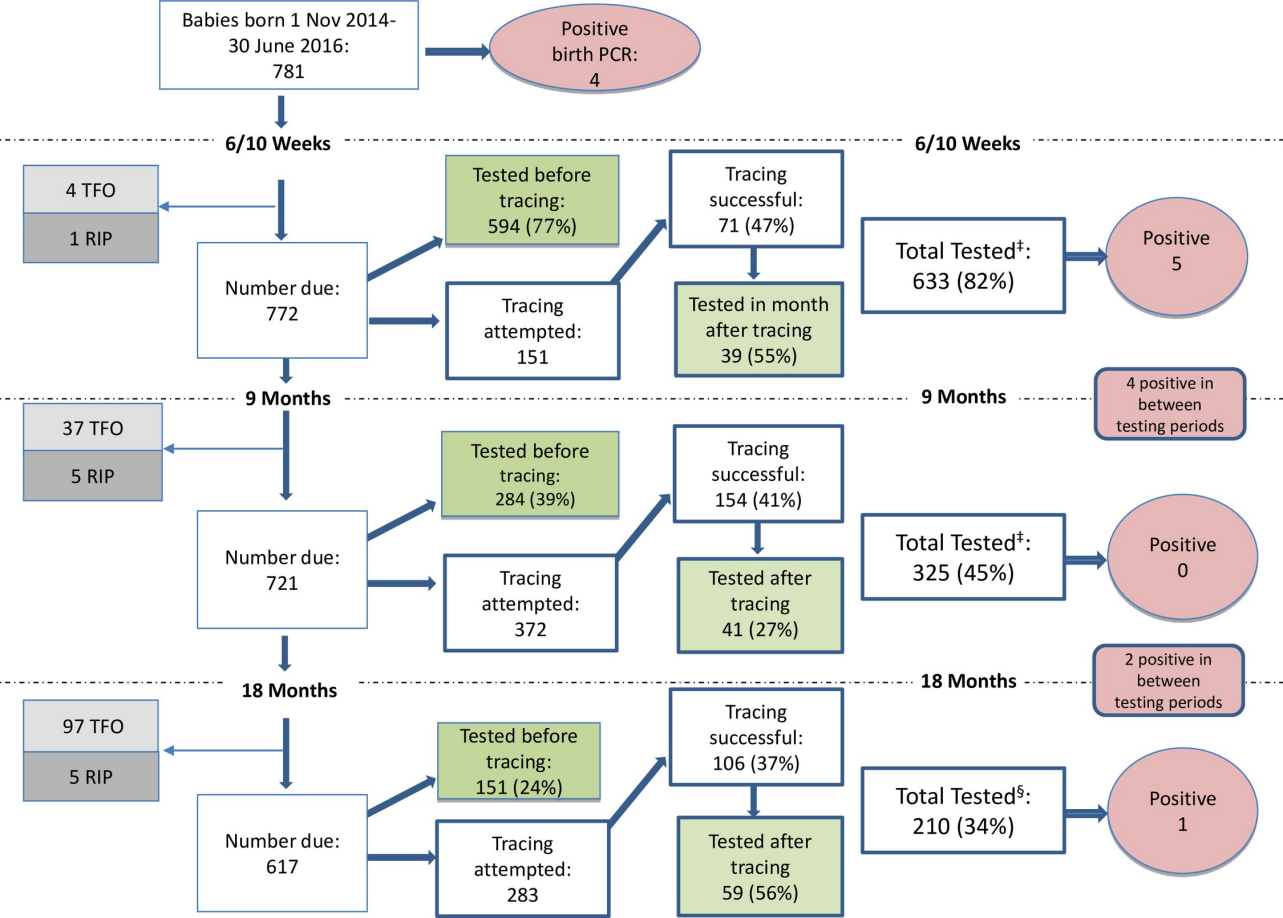
Flow chart of HIV testing post birth PCR before and after tracing. ^†^6–12 weeks + within 1 month of tracing; ^‡^8–10 months+ within 1 month of tracing; ^§^17–19 months+ within 1 month of tracing.

At 6/10 weeks, excluding TFO and deaths, 594 (77%) infants completed their routine PCR, of which 5 were positive (Fig 1). Of the 151 attempted tracing, 39 (25%) came back for testing. After tracing, 633 (82%) of infants completed their routine PCR.

After excluding TFO and deaths, at 9 months, 284 (39%) tested before tracing with no positive PCRs. Of the 372 attempted tracing, 41 (11%) tested after tracing. After tracing, 325 (45%) of infants completed their routine 9 months rapid HIV test.

After excluding TFO and deaths, at 18 months, 151 (24%) tested before tracing with one positive result. Of the 283 attempted tracing, 59 (20%) tested after tracing. After tracing, 210 (34%) of infants had completed their routine 18 months rapid HIV tests.

Six additional babies tested positive outside the testing ranges as defined above, most likely in the context of illness, with a total of 16 out of 781 babies (2%) who tested HIV positive before 20 months.

### Baseline characteristics as predictors of uptake of testing at 6/10 weeks, 9 months and 18 months

**Fig 2:**
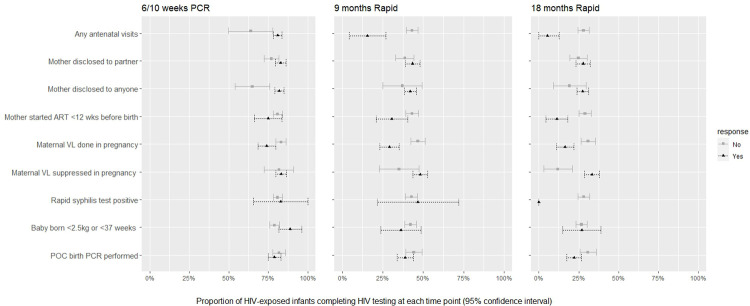
Proportion of HIV-exposed uninfected infants who completed HIV testing at each time point by baseline characteristics (95% confidence intervals).

Infant test completion was higher among those whose mothers attended antenatal care than those who did not attend antenatal care at 6/10 weeks (RD = 17%; 95%CI: 3%-31%), at 9 months (RD = 28%; 95%CI: 16%-40%), and at 18 months (RD = 23%; 95%CI: 14%-31%) (Fig 2 and Table 3). Mothers who had disclosed their HIV status to anyone at birth were more likely to test their infants at 6/10 weeks than those who had not disclosed to anyone (RD = 17%;95%CI: 6%-29%), but this effect was not clear for later tests.

**Table 3:** Comparison of percentage of infants completing 6/10 weeks HIV PCR after birth PCR or no birth PCR.

Risk Difference (95% CI)*			
	6/10 weeks PCR	9 months Rapid	18 months Rapid
**Mother disclosed to anyone**	-17.3% (-28.8 to -5.8)	-4.9% (-17.5 to 7.7)	-8.1% (-19 to 2.8)
**Mother disclosed to partner**	-6.1% (-12 to -.3)	-4.8% (-12.1 to 2.6)	-3% (-10.1 to 4.1)
**Rapid syphilis test positive**	2.7% (-14.8 to 20.1)	3.9% (-21.6 to 29.5)	-28.2% (-31.9 to -24.5)
**Maternal VL completed in pregnancy **	-9% (-15.4 to -2.5)	-17.7% (-25.1 to -10.2)	-14.4% (-21.3 to -7.5)
**Maternal VL suppressed in pregnancy **	-1.4% (-11.3 to 8.4)	-13.1% (-26.4 to .1)	-21.3% (-31.5 to -11.2)
**Mother started ART <12 wks before birth**	-6.1% (-15.4 to 3.2)	-12.5% (-23 to -2)	-17.7% (-25.7 to -9.8)
**Any antenatal visits**	17% (2.7 to 31.2)	27.7% (15.7 to 39.6)	22.6% (14.5 to 30.7)
**Baby born <2.5kg or <37 weeks**	9.2% (1.3 to 17.2)	-5.9% (-18.9 to 7)	.2% (-12.4 to 12.8)
**POC birth PCR performed**	2.8% (-2.7 to 8.4)	-5.5% (-12.6 to 1.7)	-8.7% (-15.6 to -1.8)

*Risk of test complete-risk of test incomplete

Test completion at 9 and 18 months was higher for those whose mothers started ART more than 12 weeks before birth compared to those who started ART less than 12 weeks before birth (9 months: RD = 12%; 95%CI: 2%-23%; 18 months: RD = 18%; 95%CI: 10%-26%). Mothers who had VLs completed during pregnancy were more likely to have infants tested at 6/10 weeks (RD = 9%; 95%CI:2%-15%), at 9 months (RD = 18%; 95%CI:10%-25%), and at 18 months (RD: 14%; 95%CI: 7%-21%), compared to those with missing or incomplete VLs. Among those that had viral loads, maternal VL suppression antenatally predicted infant testing at 9 months (RD: 18%; 95%CI: 10%-25%) and at 18 months (RD: 21%; 95%CI: 11%-31%). No clear association was found between RPR positivity and test uptake, possibly because of small numbers with available positive results.

Babies born prematurely were more likely to complete 6/10 weeks PCRs (RD = 9%; 95%CI: 1%-17%), but not later tests. POC testing at birth did not influence the proportion of infants testing at 6/10 weeks or 9 months, but was associated with a slightly lower 18 months test uptake (RD = 9%; 95%CI: 2%-16%).

## Discussion

In this study, we evaluated the uptake of HIV testing in HIV uninfected exposed infants in the first 18 months of life after birth PCR. We found that the uptake of 6/10 weeks testing was 77% without tracing, and 82% with tracing. When including all infants in the cascade (including transfer outs and death) and comparing to a historical cohort without birth testing, we found that infants who tested at birth were 22% more likely to have a 6/10 weeks test compared to those not tested at birth. We found no significant association between POC vs no POC testing and the likelihood of returning for the 6/10 weeks PCR test. These results combined suggests that birth PCR (with or without POC) does not have a negative impact on infants returning for the 6/10 weeks test. Uptake of HIV infant testing further along the cascade was low with only 39% and 24% of infants testing respectively at 9 months and 18 months. Tracing moderately improved their return to testing for 9 months from 39% to 45% and for 18 months from 24% to 34%. In view of the time and effort spent on tracing, it is questionable if tracing is worthwhile. The overall positivity rate for the duration of the cascade is 2%. When looking at association with baseline characteristics, we found that mothers who did not attend antenatal care were less likely to bring their child back for 6/10 weeks, 9 months and 18 months test. We also found that absence of disclosure was associated with lower uptake of the 6/10 weeks test (but not the 9 months and 18 months test uptake).

Other studies (in the same period and context) have found better uptake of the 6/10 weeks PCR, such as a reported 89.8% uptake of 6 weeks PCR [7]. In their study, Kalk et al. used very robust provincial data, from multiple sources, which could explain the improved uptake [7]. Furthermore, it is likely that the uptake of 6/10 weeks PCR has been affected by the numerous changes implemented in the PMTCT guidelines over the time period described. Of note, both studies suggest an uptake of 6/10 weeks PCR adequate enough to make birth PCR cost effective (birth PCR is only cost-effective if the 6/10 week PCR uptake is >63%) [3]. When including all infants in the cascade (including transfer outs and death) and comparing to a historical cohort without birth testing, we found that infants who tested at birth were 22% more likely to have a 6/10 weeks test compared to those not tested at birth. We found no significant association between POC vs no POC testing and the likelihood of returning for the 6/10 weeks PCR test. These results differ from those of other studies done in Cape Town. One study, done in a hospital setting, showed that performing a birth PCR significantly negatively affected the return of high risk infants for 6 week testing compared to no birth PCR (73% vs 85% return) [24]. The authors noted that half of the infants were tested because of late or no maternal antenatal ART coverage, suggesting previous suboptimal patient care-seeking behaviour. Another study, also done in Cape Town, compared results from primary and tertiary care centres (most of the birth PCRs were previously done in hospitals) and showed that 6/10 week attrition was more likely among those receiving a birth PCR compared to no birth PCR, even after adjusting for potential confounders (aOR 0.18 (0.12 to 0.26)) [7]. Our results are however in accordance with a qualitative study done in primary care settings in Lesotho suggesting that mothers are willing to come back for further testing post birth PCR [25].

Uptake of HIV infant testing further along the cascade was low with only 39% and 24% of infants testing respectively at 9 months and 18 months. These results are in line with the subdistrict level data reporting 34% uptake of the HIV test at 18 months in 2015 [18]. Looking at patient related factors, poor test uptake at 9 months and 18 months could be due to the high geographical mobility of the mothers in our context (children are often sent to the Eastern Cape province as they get older). Another contributing factor could be the high mobility between health facilities and different levels of care, leading to the infants not necessarily being recognised and tested as HIV-exposed. Furthermore, although the test might have been done, the result might not have been accessible. From a health system perspective, accessing 9 months and 18 months test results is much more time consuming and challenging than for PCR results, as it relies on physically finding folders or registers where the tests are documented versus accessing an electronic database. Proposed future solutions include digitisation of rapid HIV tests, universal 18 months testing of all infants (not only HIV-exposed ones), improving the interface between different electronic health record systems, as well as unique identifier numbers. Other potential solutions also include improving the implementation of integrated care in the postnatal period, for example using the postnatal club model [26]. The postnatal club is an example of a differentiated model of care offering peer-led psychosocial support and comprehensive integrated care to all HIV-positive mothers and their HIV-exposed infants [27, 28].

Tracing improved the uptake of 9 months testing from 39% to 45% and 18 months testing from 24 to 34%. This small impact may have several explanations. First of all, due to the high geographical mobility of lower income populations, such as the one in Khayelitsha, contact details frequently change [29, 30]. This means that it is often difficult to tease out if the patients are lost to follow-up or if they have just moved away [31, 32]. Considering the amount of time and effort spent on the tracing activities, tracing needs to be adapted to cater to the population served and is unlikely sufficient by itself. Other interventions such as the peer mentor support provided by mentor mothers might be more suited to mother-infant dyads [33], and electronic medical records can help make tracing more efficient [34].

The overall positivity rate for the duration of the cascade is 2%. Our results are in line with a more recent study looking at the same population which found MTCT of 1.8% at 12 months old [35], and lower than the national average of 4.3%. Our study positivity rate could be an underestimate because of transfer outs, potential silent transfers, and LTFU [36]. It is also possible that the Western Cape has lower 18 months positivity rate than the national one.

When looking at baseline characteristics, mothers who did not attend antenatal care were less likely to bring their child back for 6/10 weeks, 9 months and 18 months test. We also saw in this study that absence of disclosure was associated with lower uptake of the 6/10 weeks test (but not the 9 months and 18 months test uptake). However, it should be noted that disclosure was only assessed at baseline and not subsequently and might have changed over time. Women who demonstrate poor personal health seeking behaviour seem to show similar trends when caring for their children. Reasons for this poor health seeking behaviour could be multiple and might include mental health issues, substance use as well as absence of disclosure or fear of stigma [37–39]. More qualitative research is needed to better understand the reasons for these women not engaging in care. In an era when we are working towards eMTCT, it is vital to focus specifically on this hard to reach population as they constitute a high risk factor for MTCT [40]

### General limitations

A limitation of our baseline maternal data was the large proportion of missing ARV status. Their ARV status at birth would be important in understanding the risk of MTCT to their children. However, we can infer from another study done in the same setting and at similar times that over 90% of them would have been on ARVs [35]. In addition, if the mother had attended antenatal care at a different clinic, it may have been missed.

During our study, when mothers of infants who were tested at birth were given the PCR result, they were also counselled on the necessity of returning for follow-up HIV testing and were given a date for their next PCR test. This could have increased their uptake of the 6/10 weeks test. However, apart from the birth PCR and counseling described, this study is very representative of the real life context as the rest of the testing cascade unfolded without any specific interventions or incentives. Only infants who were not tested following standard of care protocols were actively traced. Uptake of 6/10 weeks PCR may have been affected by the many changes in the PMTCT guidelines between 2015–2018 as it could have led to confusion for the mothers/caregivers and for the health care workers. Testing at 9 months and 18 months were not affected by these guideline changes.

Another limitation is that when comparing the 6/10 weeks PCR result for the birth PCR and no birth PCR groups, we compared our study cohort to a historical control group. Collection of information was different for the historical control group as we collected information from labour ward register, whereas for our study participants, we actively filled a baseline questionnaire form per mother-infant dyad. In addition, the laboratory electronic system changed over that time, making collection of PCR information more difficult for the historical group. The extra counselling the birth PCR group received could have also positively influenced their return for further infant testing.

Other potential biases include the fact that the population studied excluded mothers who were transferred to secondary care hospitals, potentially higher risk (medically and socio-economically). We could not check any laboratory results outside the Western Cape Province so are unable to account for “silent transfers”. Furthermore, although we could verify PCRs done in any primary care facilities in the Western Cape, we were not able to check for HIV rapid test results in hospital settings. These factors could have contributed to the low test uptake at 9 months and 18 months. Of note, outcomes of death and transfer outs were reported by primary caregivers and not verified. Given that women residing in Khayelitsha are very mobile, over time, changes in phone numbers and addresses become increasingly likely, leading to increased loss to follow-up as well as to the possibility of geographical relocation. Although tracing increased the uptake of testing at 9 months and 18 months, these challenges are huge limitations to the effectiveness of tracing.

## Conclusions

Uptake of HIV testing for HIV-exposed uninfected infants in the first 18 months of life shows good completion of the 6/10 weeks PCR but suboptimal uptake of HIV testing at 9 months and 18 months, despite tracing efforts. Birth PCR testing did not negatively affect uptake of the 6/10 weeks HIV test compared to no birth PCR testing. Lack of maternal antenatal care attendance appeared to be a predictor of lower uptake of HIV testing for the infants throughout the whole cascade. Implementation of innovative solutions for the postnatal period, such as the integrated model of care PNC, are needed to retain in care mothers and their children.

## Supporting information

S1 File Field validation of point of care PCR machine [41]. (DOCX)

S2 File Case report form (version April 2016). (DOCX)
