# Supplementary material for: An analysis of the HIV testing cascade of a group of HIV-exposed infants from birth to 18 months in peri-urban Khayelitsha, South Africa
Source: PLoS One. 2022 Jan 14;17(1):e0262518. doi: 10.1371/journal.pone.0262518 (PMC8759686; doi:10.1371/journal.pone.0262518)
Supplement: S1 File — (DOCX) [file pone.0262518.s001.docx]

**SUPPLEMENTAL FILE 1: Field validation of Point of Care PCR machine**

1. Sample collection

Whole blood samples were collected in EDTA microcontainers via heel prick of the newborn, and if this failed via venesection. Some infants were co-recruited into the INFANT study , and if so, blood samples would be collected as per INFANT protocol [41]. 25uL was removed with a capillary tube and inserted into the disposable POC cartridge. The rest of the sample was sent to the central laboratory for testing via routine NHLS sample transport from the clinic. The laboratory electronically informed the clinical team of all PCR results done, including indeterminate, as soon as results were available.

ii. POC and maintenance

Two POC machines were purchased from Alere and were installed at the clinic in July 2015, after laboratory validation. The manufacturer provided initial training on the platform for all staff using the machines and provided technical support for any breakdown in the device. If a faulty platform had to be sent to Germany for repair, a substitute machine would be lent temporarily. No Alere Q cartridges were available between March and April 2016 due to supply issues from the manufacturer.

iii. Negative results

Negative results on POC were communicated to the mothers as soon as they were available. Laboratory-based negative results were communicated to the mothers when they would come back to the primary care clinic for their first follow-up visit after birth by the research nurse. In both instances, the research nurse would also counsel the patient on the need for further infant testing and give her a date for the 6/10 weeks PCR test.

iv. Positive results

If a positive result was obtained on the laboratory-based PCR, the mother was called back to the clinic to be informed of the result, a confirmatory viral load was sent and the infant initiated on ART, as per local guidelines [21].

If a positive result was obtained on POC, it was confirmed with a laboratory-based positive result. As per the protocol, a positive result on POC was acted on immediately (before confirmation), and a primary care medical officer (available seven days of the week) called in to initiate the infant on antiretroviral therapy, as per national guidelines. In both instances, the mother was counseled by the research nurse, according to counseling sessions developed by the MSF patient support team.

v. Indeterminate and error results

If an indeterminate result occurred in the laboratory, mother was recalled for a repeat blood sample draw and counseling given.

If one POC test resulted in an error message, the sample would be run on the second POC machine. If a repeat error message occurred, a repeat specimen would be collected from the infant and re-run in the first machine. The mother was counseled appropriately.
