# Supplementary figures and images for: An analysis of the HIV testing cascade of a group of HIV-exposed infants from birth to 18 months in peri-urban Khayelitsha, South Africa
Source: PLoS One. 2022 Jan 14;17(1):e0262518. doi: 10.1371/journal.pone.0262518 (PMC8759686; doi:10.1371/journal.pone.0262518)

**SUPPLEMENTAL FILE 2: Case Report Form (Version April 2016)**


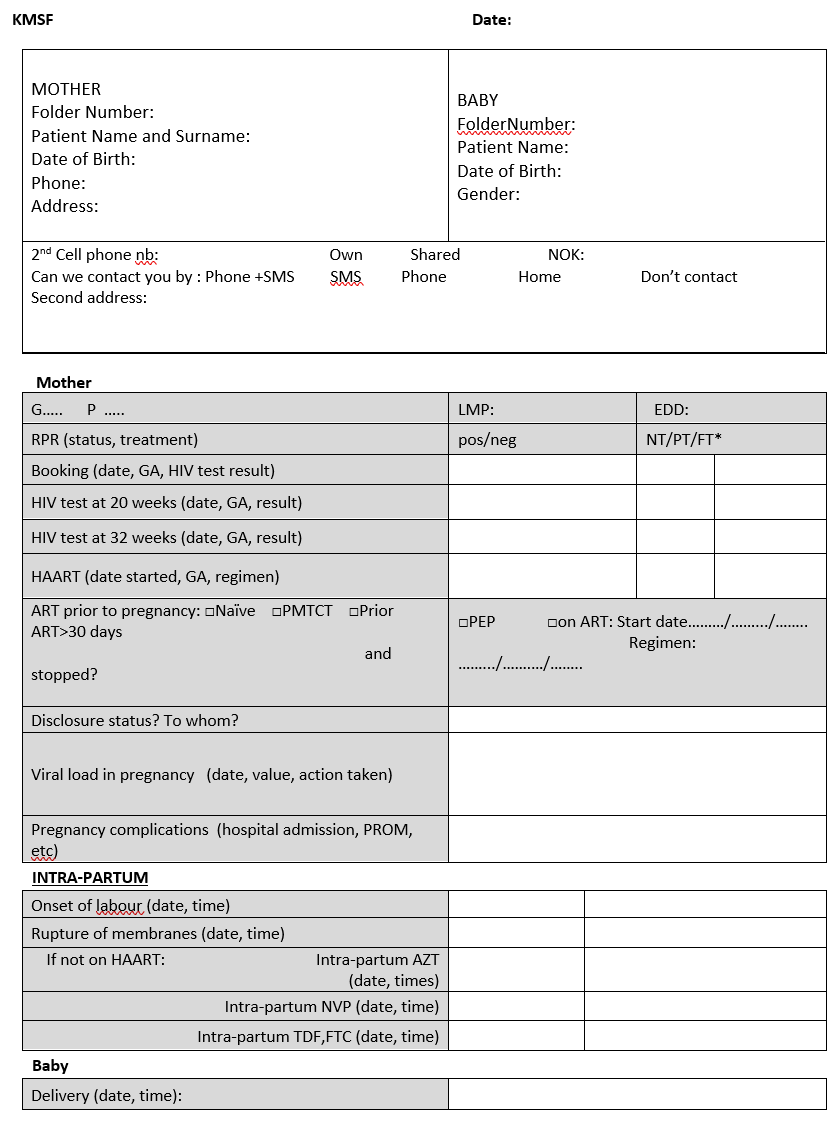


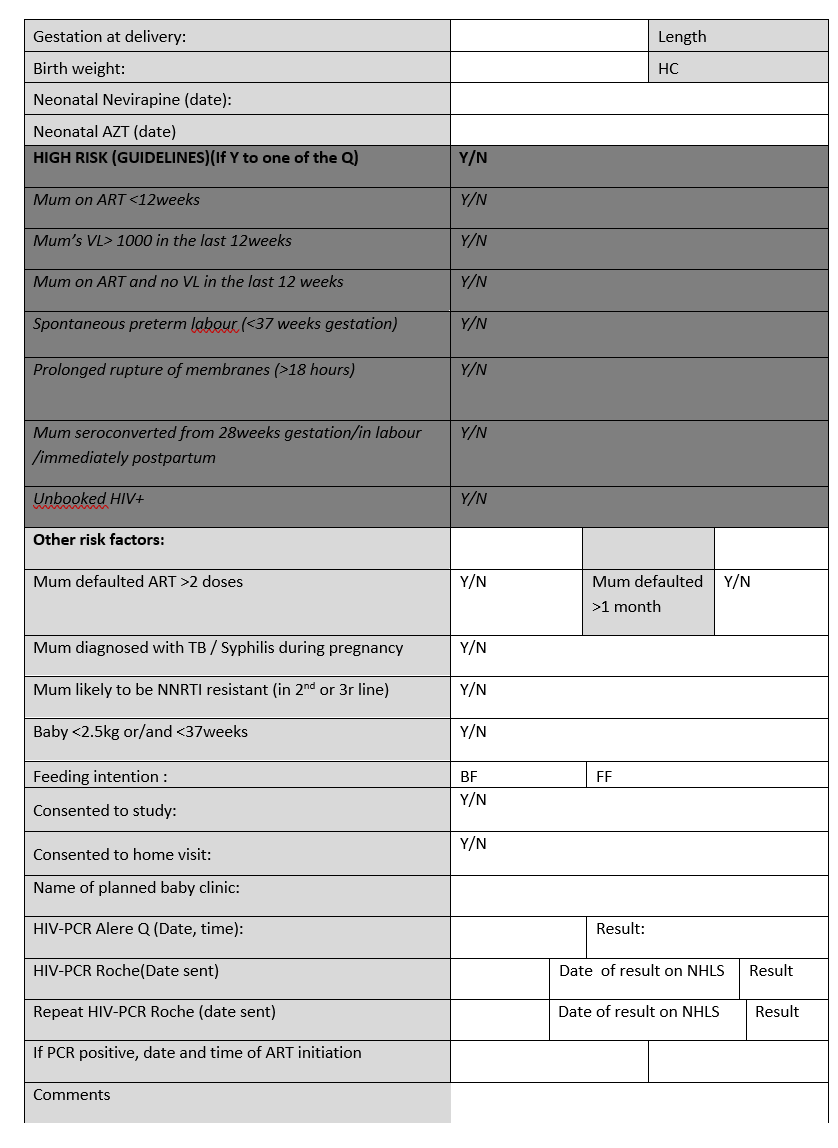

Supplement: S2 File — (DOCX) [file pone.0262518.s002.docx]
